# Effects of Replacing Fishmeal with Enzymatically Hydrolyzed Pork Bone Meal (EHPBM) on Growth, Antioxidant Capacity, and Nutritional Metabolism in *Micropterus salmoides*

**DOI:** 10.3390/ani15233359

**Published:** 2025-11-21

**Authors:** Xinlan Bai, Haifeng Mi, Dongyu Huang, Hualiang Liang, Wu Shan, Mingchun Ren, Lu Zhang, Tao Teng

**Affiliations:** 1 Wuxi Fisheries College, Nanjing Agricultural University, Wuxi 214081, China; 2 Tongwei Agricultural Development Co., Ltd., Key Laboratory of Nutrition and Healthy Culture of Aquatic, Livestock and Poultry, Ministry of Agriculture and Rural Affairs, Healthy Aquaculture Key Laboratory of Sichuan Province, Chengdu 610093, China; 3 Key Laboratory of Integrated Rice-Fish Farming Ecology, Ministry of Agriculture and Rural Affairs, Freshwater Fisheries Research Center, Chinese Academy of Fishery Sciences, Wuxi 214081, China; 4 JIANGSU MASUN BIO-TECH., LTD, Wuxi 214126, China

**Keywords:** enzymatically hydrolyzed pork bone meal (EHPBM), fishmeal, metabolism

## Abstract

In this 8-week feeding trial, we systematically investigated the effects of replacing fishmeal with enzymatically hydrolyzed pork bone meal (EHPBM) at graded inclusion levels (EHPBM0, EHPBM20, EHPBM50, and EHPBM100) in largemouth bass (*Micropterus salmoides*). The results showed that the EHPBM50 group maintained growth performance comparable to the fishmeal-based control, whereas higher replacement levels led to significant metabolic disturbances. Specifically, the EHPBM100 group exhibited marked reductions in final body weight (FBW), weight gain rate (WGR), and specific growth rate (SGR), along with an elevated feed conversion ratio (FCR). Serum biochemical markers—alanine aminotransferase (ALT) and aspartate aminotransferase (AST)—were significantly decreased in a dose-dependent manner under EHPBM50 and EHPBM100 substitution. Regarding whole-body composition, ash content was significantly lower in the EHPBM50 group, while no significant differences were observed in other metrics compared to the control. Furthermore, dietary EHPBM inclusion enhanced systemic antioxidant capacity. All EHPBM substitution groups showed significantly increased superoxide dismutase (SOD) and catalase (CAT) activities, along with significantly reduced malondialdehyde (MDA) levels. In key metabolic pathways, compared with the EHPBM0 group, the expression of *mtor* and *rps6k* genes was significantly up-regulated in the EHPBM50 group, while that of *g6pase*, *fbp1*, and *cpt1* genes was significantly down-regulated. Intestinal integrity markers (*occludin*, *zo-1*) and nutrient transporters (*pept1*, *lat1*) remained largely unaffected except in the EHPBM100 group, indicating the species’ tolerance to partial fishmeal replacement. In summary, these findings demonstrate that EHPBM can effectively replace up to 50% of fishmeal in largemouth bass feed without compromising growth performance or nutrient utilization, while significantly enhancing antioxidant capacity.

## Simple Summary

This study aimed to investigate the effects of replacing fish meal with enzymatically hydrolyzed pork bone meal (EHPBM) on the growth, antioxidant capacity, and nutritional metabolism of largemouth bass (*Micropterus salmoides*). The results indicated that EHPBM could replace up to 50% of the fish meal without any significant adverse effects on growth. Furthermore, the inclusion of EHPBM significantly enhanced the fish’s antioxidant capacity without significantly impacting liver health. However, excessively high substitution levels may induce intestinal inflammation and disrupt nutritional metabolism.

## 1. Introduction

Fishmeal has long been a conventional protein source in animal feeds [1]. In recent years, the continuous expansion of aquaculture [2] has led to a surge in fishmeal demand, driving up its market price [3]. This cost increase has posed formidable challenges to the sustainable development of the industry [4]. Consequently, seeking viable alternatives to fishmeal has become an imperative trend, driven by the dual pressures of expanding aquaculture scales and diminishing fishmeal resources [5]. Protein sources serving as fishmeal alternatives are primarily categorized into plant-based and animal-based origins. Plant-derived proteins face multiple constraints in aquaculture applications [6,7]. Furthermore, they often lack certain essential amino acids critical for fish nutrition [8]. In contrast, animal proteins offer a more balanced amino acid profile along with beneficial compounds, making them stronger candidates to replace fishmeal [9]. Compared with plant-based proteins, animal protein sources contain more proteinogenic amino acids (AAs), with generally superior amino acid ratios and higher digestibility [10]. For carnivorous fish, the higher content of non-essential amino acids (NEAAs) in animal protein [11] can reduce the energy required for de novo synthesis of these amino acids, as well as the consumption of essential amino acids. This contributes to improved feed utilization efficiency in carnivorous fish [12]. Terrestrial animal byproducts are particularly promising as fishmeal substitutes due to their availability and cost benefits [13].

Meat and bone meal (MBM), including pork bone meal (PBM), is a rendered by-product derived from the meat processing industry [14]. It consists of rendered, partially defatted residual materials obtained after being excluded from the human food supply chain [15]. MBM not only has high protein content but also provides abundant mineral phosphorus [16]. Furthermore, studies have shown that MBM serves as a valuable source of non-essential amino acids [17]. However, the use of unprocessed plant or animal proteins can hinder nutrient absorption in aquatic species [18,19]. Although MBM is rich in protein, its unprocessed form significantly limits its inclusion level in feed formulations [20]. Moreover, untreated MBM not only reduces apparent nutrient digestibility in largemouth bass (*Micropterus salmoides*) [21] but also adversely affects growth performance and feed utilization efficiency [22]. In white shrimp (*Litopenaeus vannamei*), replacing more than 25% of fishmeal with untreated MBM has been shown to significantly compromise growth performance [23]. The large-molecular-weight proteins in untreated MBM limit its digestion and absorption in fish, while enzymatic hydrolysis can convert these proteins into small peptide molecules that are more readily digestible and absorbable. Processing methods such as enzymatic hydrolysis and fermentation could effectively enhance feed utilization efficiency and improve nutrient digestion and absorption, representing a promising direction worthy of further in-depth investigation.

Enzymatic hydrolysis is a widely used protein modification technique that significantly enhances nutrient bioavailability [24,25]. This process improves the digestibility of the resulting products [26], enriching the material with bioactive peptides and free amino acids [27], while also improving feed quality and palatability [28]. Due to its straightforward reaction process and mild operating conditions, enzymatic hydrolysis is extensively applied in the food processing industry [29]. For example, the addition of 8% enzymatically hydrolyzed chicken paste (ECP) significantly promoted the growth and health of sea cucumber (*Apostichopus japonicus*) fed a low-fishmeal diet [30], while 1% enzymatically hydrolyzed black soldier fly paste (ZBSFP) was identified as a suitable additive for partially replacing fishmeal in largemouth bass feed [31]. Similarly, replacing 12.4% of fishmeal with enzymatic hydrolysate poultry by-product meal had no significant impact on the growth performance of juvenile largemouth bass [32]. In rohu (*Labeo rohita*), diets containing poultry by-product meal (PBM) supplemented with protease improved growth performance within a defined threshold [33]. Furthermore, bio-processed poultry by-product meal has been shown to fully replace fishmeal protein in Nile tilapia diets without adverse effects [34]. Additionally, the inclusion of MBM also influences fish nutritional metabolism. Increased dietary MBM has been shown to adversely affect lipid utilization in juvenile snakehead (*Channa argus*) [35]. In contrast, feeding MBM did not significantly affect fat deposition in juvenile hybrid striped bass (*Morone chrysops* × *M. saxatilis*) [36]. However, incorporation of over 20% MBM increased hepatic lipid deposition in gilthead seabream (*Sparus aurata*) [37]. Meanwhile, MBM supplementation reduced the efficiency of protein synthesis and deposition in European eel (*Anguilla anguilla*) [38]. Based on the aforementioned findings, the potential of enzymatically hydrolyzed pork bone meal (EHPBM) as a fishmeal substitute warrants further investigation. It remains to be thoroughly explored whether EHPBM supplementation can improve feed quality, enhance nutrient absorption efficiency, and increase overall feeding value. It may be inferred that partial replacement of fishmeal with EHPBM in largemouth bass feed would not adversely affect growth performance and might even enhance immune and antioxidant capacities to some extent.

Largemouth bass has gained widespread popularity in freshwater aquaculture owing to its rapid growth rate and high economic value [39]. As a carnivorous species, it exhibits high dietary protein requirements [18,39,40]. Although previous studies have explored fishmeal substitution using various enzymatic hydrolysates in largemouth bass [30,31,32,33], systematic research on enzymatically hydrolyzed pork bone meal (EHPBM) as a fishmeal alternative remains limited. Therefore, this study aims to identify the optimal substitution ratio of EHPBM for fishmeal and evaluate its subsequent effects on nutritional metabolism pathways in juvenile largemouth bass, thereby contributing to reduced reliance on fishmeal.

## 2. Materials and Methods

### 2.1. Diet Preparation

As the addition of different accounts of EHPBM (supplied by JIANGSU MASUN BIO-TECH., LTD (Wuxi, China)), four groups were designed in this study. These four groups replaced 0, 20, 50, and 100 percent of fishmeal. The detailed feed formulations are presented in Table 1. According to the formulations, all ingredients were thoroughly mixed with water and oil, and then processed into extruded feed pellets with a diameter of 2.0 mm using a feed pelletizer (TSE65, YangGong Technology, Beijing, China). The pelleted feed was air-dried in a well-ventilated area. After complete drying, the feed was packed in sealed bags and stored for subsequent use. The ingredient and nutrient compositions of fish meal and EHPBM are shown in Table 2.

### 2.2. Experimental Management

The experiment was conducted using an indoor recirculating aquaculture system (RAS) at the Freshwater Fisheries Research Center, Chinese Academy of Fisheries Sciences (CAFS). The RAS, supplied by Recycling Water Aquaculture System Co., Ltd. (Qingdao, Shandong Province, China), was equipped with water purification and temperature control functions. Prior to the experiment, largemouth bass were acclimated in the system for two weeks. Juvenile largemouth bass with an initial average body weight of 6.60 ± 0.01 g were then randomly selected for the formal trial. The experiment employed a total of 16 tanks (270 L each), with four tanks assigned to each dietary group and 20 fish stocked per tank. Tank allocation followed a completely randomized design. The fish were fed twice daily at 8:00 and 16:30 to apparent satiety. Throughout the 8-week trial, water quality parameters in the indoor recirculating aquaculture system were maintained as follows: dissolved oxygen > 6.0 mg/L, temperature 28 ± 2 °C, total ammonia nitrogen < 0.1 mg/L, pH 7.0–7.8, and a photoperiod of 12 h light:12 h dark.

### 2.3. Sample Collection

Prior to formal sampling, largemouth bass across all 16 tanks were fasted for 24 h. The total biomass in each tank was weighed. Three fish were randomly selected from each tank for blood collection. The blood samples were centrifuged at 3500 rpm for 10 min at 4 °C, and the supernatant serum was stored for subsequent analysis. Liver and intestinal tissues were then collected for antioxidant capacity assessment. Additionally, liver tissue samples from one randomly selected fish per tank were fixed in 4% paraformaldehyde solution for histopathological examination. Finally, three fish per tank were randomly chosen for whole-body composition analysis.

### 2.4. Chemical Analysis

The whole-body composition of fish and feed was analyzed for four indices—ash, crude protein, moisture, and crude lipid—following the standard AOAC methods [41]. Samples were first oven-dried at 105 °C to constant weight. Crude protein content was determined using the Kjeldahl method with a K1100 analyzer (Haineng Instrument Co., Ltd., Jinan, China). Crude lipid content was measured by Soxhlet extraction on an automated system (SOX606, same manufacturer), and ash content was quantified by combustion in a muffle furnace (XL-2A, Zhuochi Instrument, Hangzhou, China) at 560 °C for 6 h.

Plasma biochemical parameters were analyzed using a Mindray BS-400 analyzer (Shenzhen, China) according to previously described procedures [42]. The measured indicators included total cholesterol (TC), alanine aminotransferase (ALT), triglycerides (TG), glucose (GLU), and aspartate aminotransferase (AST). Liver antioxidant indices—superoxide dismutase (SOD), glutathione (GSH), catalase (CAT), glutathione peroxidase (GSH-Px), and malondialdehyde (MDA)—were assessed using commercial assay kits (Nanjing Jiancheng, Nanjing, China) according to the manufacturer’s instructions.

### 2.5. Histopathology of the Liver

Liver tissue sections were first dewaxed in xylene and rehydrated through a graded ethanol series (100%, 95%, 70%). After rinsing under running tap water, the sections were immersed in a pretreatment solution for 1 min. Following pretreatment, the sections were stained sequentially with hematoxylin and eosin (H&E). The stained sections were then dehydrated through a reverse ethanol gradient (70%, 95%, 100%), cleared in xylene, mounted with resinous medium, and examined under a light microscope (Eclipse Ci-L, Nikon, Japan) at various magnifications.

### 2.6. Gene Expression Analysis

Table 3 lists the primer sequences used in this experiment. Total RNA was extracted from largemouth bass liver tissue using RNA extraction reagents from Vazyme (Nanjing, China). After quantification of the isolated RNA, real-time PCR (RT-PCR) was performed with a One Step Kit (Vazyme, Nanjing, China) on a CFX96 Touch system (Bio-Rad, Hercules, CA, USA). The PCR protocol consisted of reverse transcription at 50 °C for 3 min, initial denaturation at 95 °C for 30 s, followed by 40 cycles of denaturation at 95 °C for 10 s and annealing/extension at 60 °C for 30 s. Consistent with our previous study [43], the *gapdh* gene, which showed stable expression, was selected as the reference gene to ensure reliable and accurate quantification of gene expression. Standard curve construction followed the method described in our earlier work [44].

### 2.7. Data Analysis

For the one-way ANOVA F-test, G Power analysis determined a minimum sample size of 180, assuming an effect size of 0.25, α = 0.05, and statistical power of 0.8. Our experimental design meets this requirement. Then, normality and equal variance were used to analyze all data. Before analysis, the normality of data distribution and homogeneity of variance were assessed using the Shapiro–Wilk test and Levene’s test, respectively. The data were shown as mean ± standard error. Using SPSS (22.0) for one-way ANOVA. Post hoc pairwise comparisons were performed using Tukey’s honestly significant difference (HSD) test. Distinct superscript letters assigned to the same measured parameter denote statistically significant differences (*p* < 0.05).

## 3. Results

### 3.1. Growth Performance

Table 4 provides growth performance results. When fishmeal was replaced with 50 percent, no significant effects were observed on final body weight (FBW), feed conversion ratio (FCR), weight gain rate (WGR), and specific growth rate (SGR) (*p* > 0.05). However, the EHPBM100 group significantly reduced FBW, WGR, and SGR, while concurrently increasing FCR (*p* < 0.05). These groups showed no significant impact on survival rate (*p* > 0.05).

### 3.2. Whole Body Composition

Results can be seen in Table 5. At the replacement of 100 percent of fishmeal, whole-body moisture content increased significantly (*p* < 0.05), whereas no significant differences were observed in this parameter between the groups of EHPBM20 and EHPBM50, and EHPBM0 (*p* > 0.05). EHPBM100 significantly reduced whole-body crude lipid content (*p* < 0.05). However, the groups of EHPBM20 and EHPBM50 showed no significant effects (*p* > 0.05). Whole-body ash content decreased significantly at the group of EHPBM50 and EHPBM100 substitution rates (*p* < 0.05), whereas the group of EHPBM20 fish meal replacement level had no significant impact (*p* > 0.05). Additionally, EHPBM substitution exerted no significant effects on whole-body crude protein content (*p* > 0.05).

### 3.3. Plasma Biochemistry

**Figure 1:**
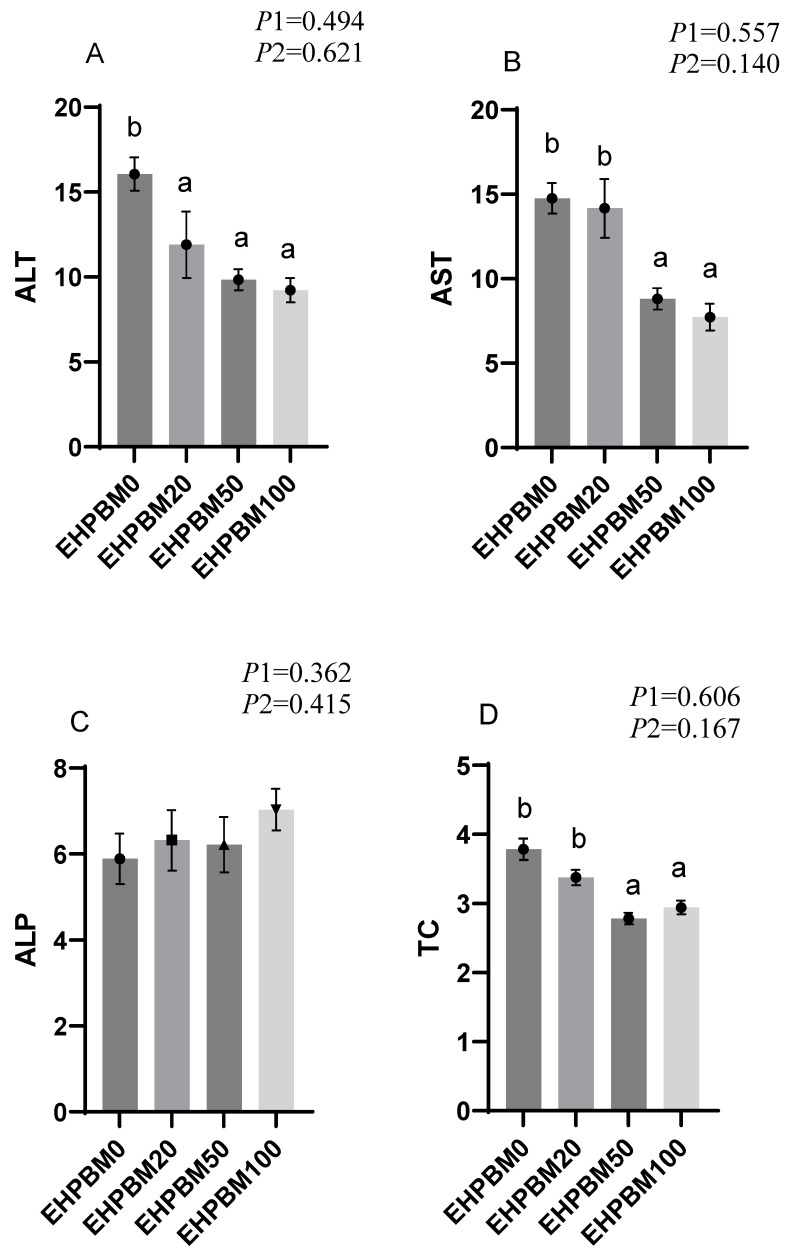

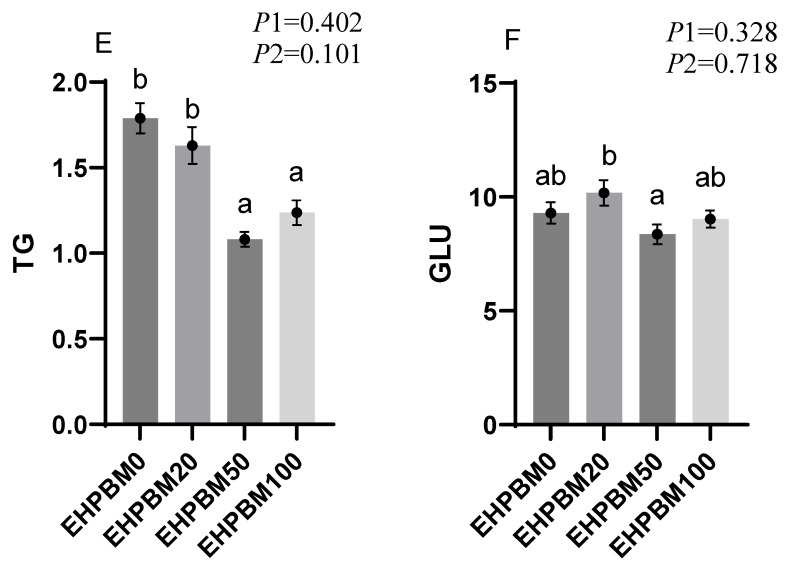
Plasma biochemistry. Statistical significance between groups is indicated by differing superscripts. (**A**) ALT; (**B**) AST; (**C**) ALP; (**D**) TC; (**E**) TG; (**F**) GLU. *p*1: the average of the *p*-values from the Shapiro–Wilk tests for each group; *p* > 0.05 indicates that the data follow a normal distribution. *p*2: *p*-value of the Levene’s test; *p* > 0.05 indicates that the data meet the assumption of homogeneity of variances.

The results of plasma biochemistry can be seen in Figure 1. Both EHPBM50 and EHPBM100 groups significantly reduced AST levels (*p* < 0.05). Similarly, TC and TG levels of these two groups were also markedly decreased compared to the control group (*p* < 0.05). However, serum GLU levels showed no significant differences compared to the control group (*p* > 0.05). Notably, EHPBM substitution significantly lowered serum ALT levels across replacement levels (*p* < 0.05).

### 3.4. Hepatic Enzymatic Indices

As illustrated in Table 6, dietary substitution of fish meal with EHPBM significantly enhanced hepatic SOD and CAT activities (*p* < 0.05), concurrently reducing MDA levels (*p* < 0.05). Notably, the EHPBM100 group markedly increased GSH content and GSH-Px activity (*p* < 0.05). Furthermore, hepatic GSH-Px activity was also significantly elevated in EHPBM50 and EHPBM100 groups (*p* < 0.05).

### 3.5. Protein Metabolism

**Figure 2:**
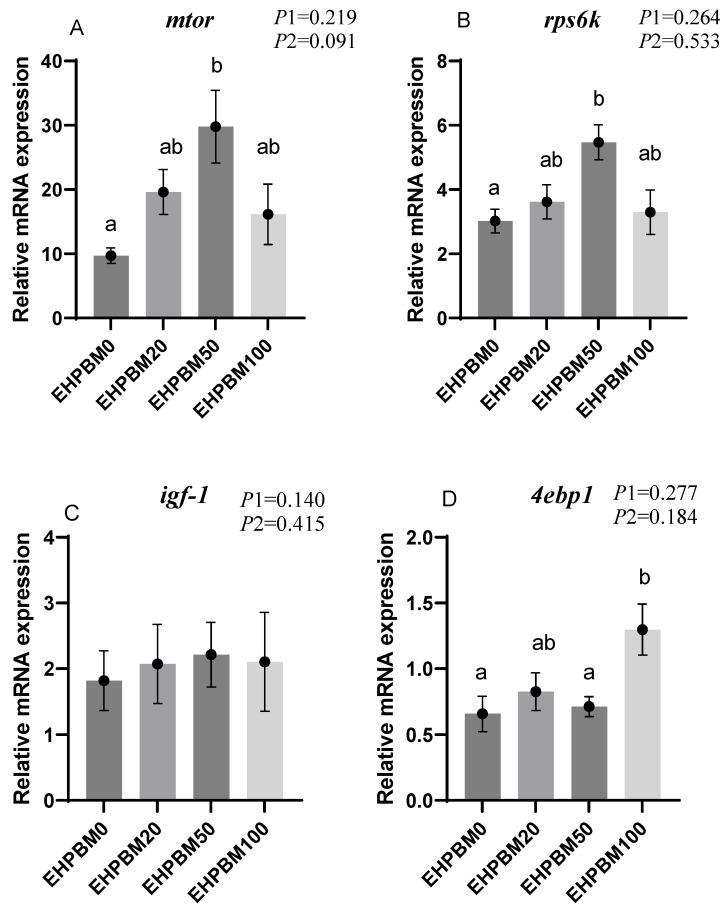
Expression of genes related to hepatic protein metabolism. Note: Statistical significance between groups is indicated by differing superscripts. (**A**) *mtor*; (**B**) *rps6k*; (**C**) *igf-1*; (**D**) *4ebp1*. *p*1: the average of the *p*-values from the Shapiro–Wilk tests for each group; *p* > 0.05 indicates that the data follow a normal distribution. *p*2: *p*-value of the Levene’s test; *p* > 0.05 indicates that the data meet the assumption of homogeneity of variances.

Figure 2 presents expression profiles of four protein metabolism-related genes under different substitution levels of EHPBM replacing fishmeal. In the group of EHPBM50, significant upregulation is observed in the expression of *mtor* and *rps6k* compared with the EHPBM0 group (*p* < 0.05). In the EHPBM100 group, the expression level of *4ebp1* shows marked upregulation compared with the EHPBM0 group (*p* < 0.05). Notably, EHPBM supplementation demonstrates no significant regulatory effect on *igf-1* expression (*p* > 0.05).

### 3.6. Glucose Metabolism

**Figure 3:**
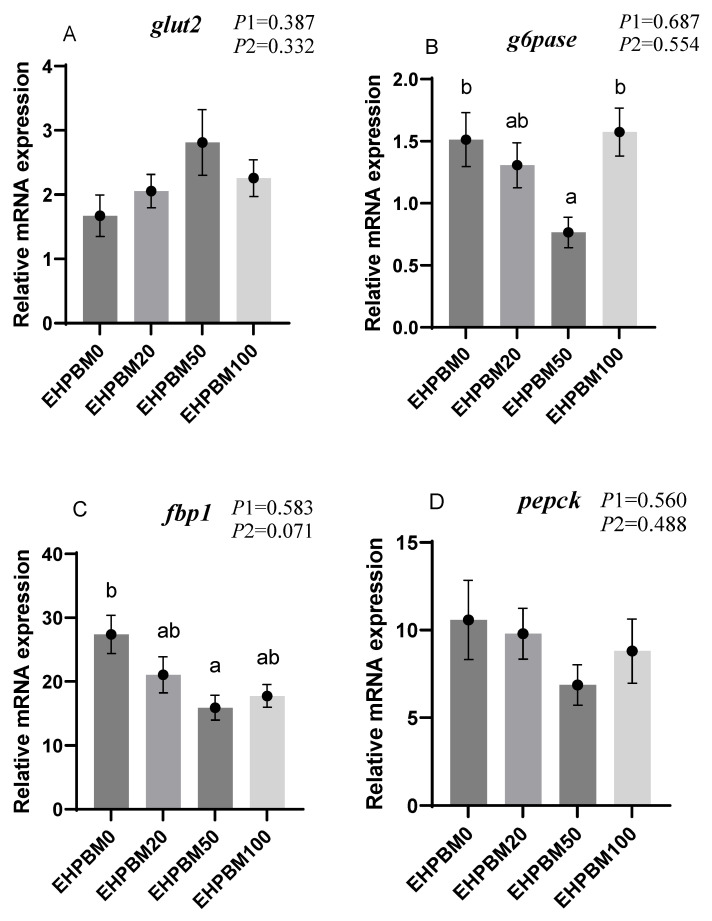

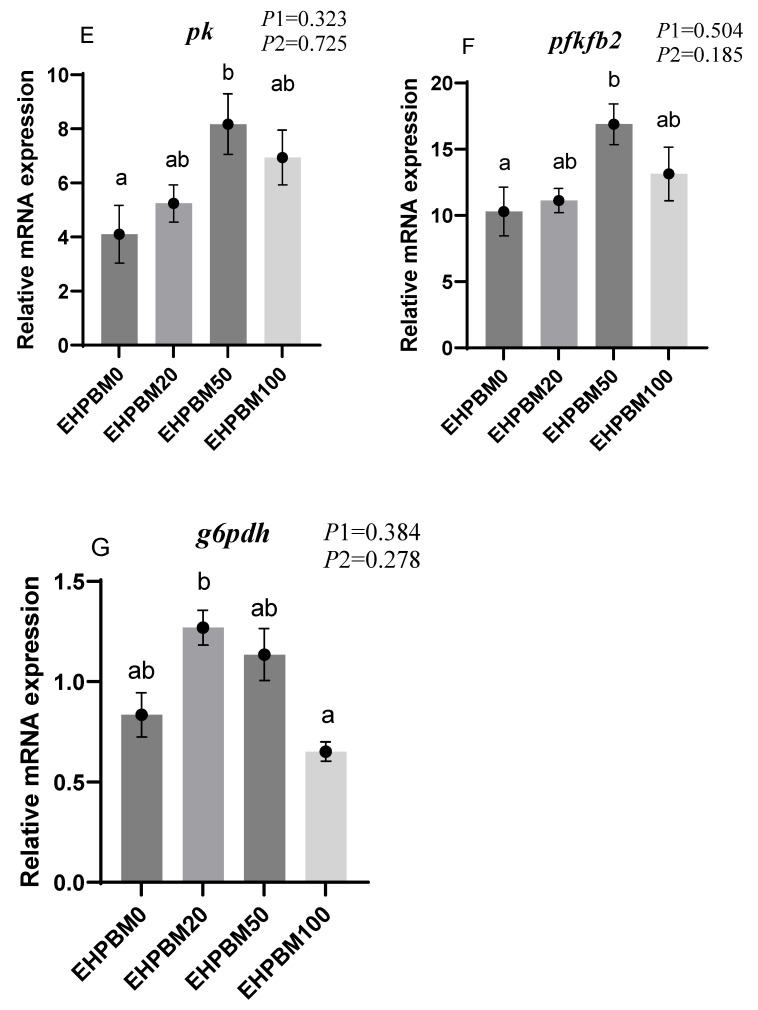
Expression of genes related to hepatic glucose metabolism. Note: Statistical significance between groups is indicated by differing superscripts. (**A**) *glut2*; (**B**) *g6pase*; (**C**) *fbp1*; (**D**) *pepck*; (**E**) *g6pdh*; (**F**) *pk*; (**G**) *pfkfb2*. *p*1: the average of the *p*-values from the Shapiro–Wilk tests for each group; *p* > 0.05 indicates that the data follow a normal distribution. *p*2: *p*-value of the Levene’s test; *p* > 0.05 indicates that the data meet the assumption of homogeneity of variances.

Figure 3 presents the expression profiles of glucose metabolism-related genes under varying substitution levels of EHPBM replacing fishmeal. In the EHPBM50 group, *g6pase* and *fbp1* exhibited significant downregulation, while *pk* and *pfkfb2* demonstrated marked upregulation compared with the EHPBM0 group (*p* < 0.05). Furthermore, compared to the control group, EHPBM supplementation revealed no significant alterations in *glut2*, *pepck*, or *g6pdh* expression levels (*p* > 0.05).

### 3.7. Lipid Metabolism

**Figure 4:**
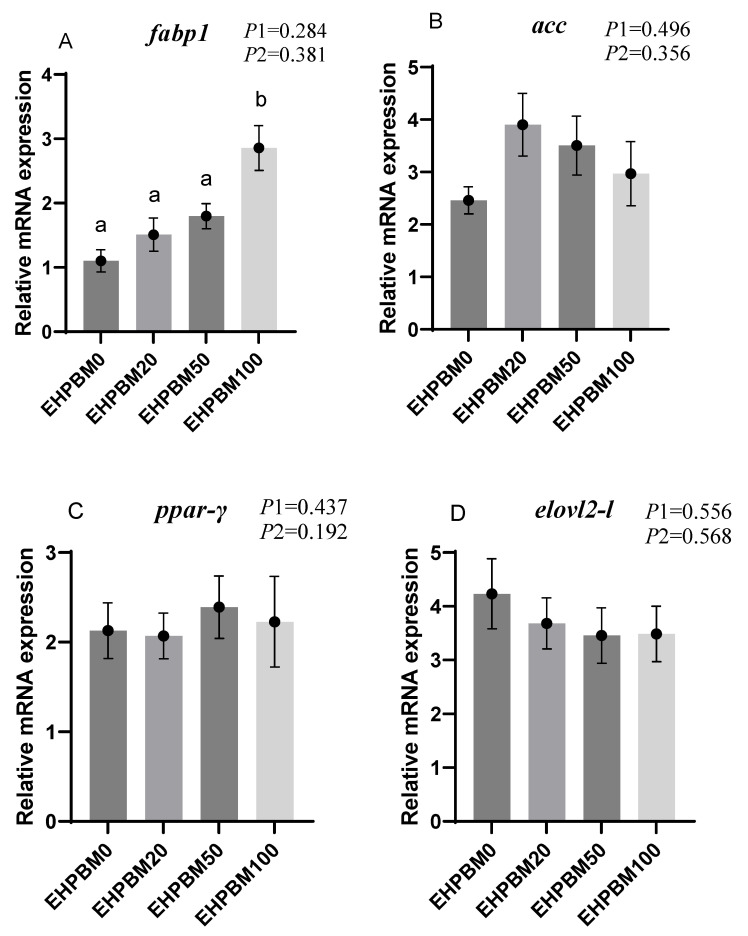

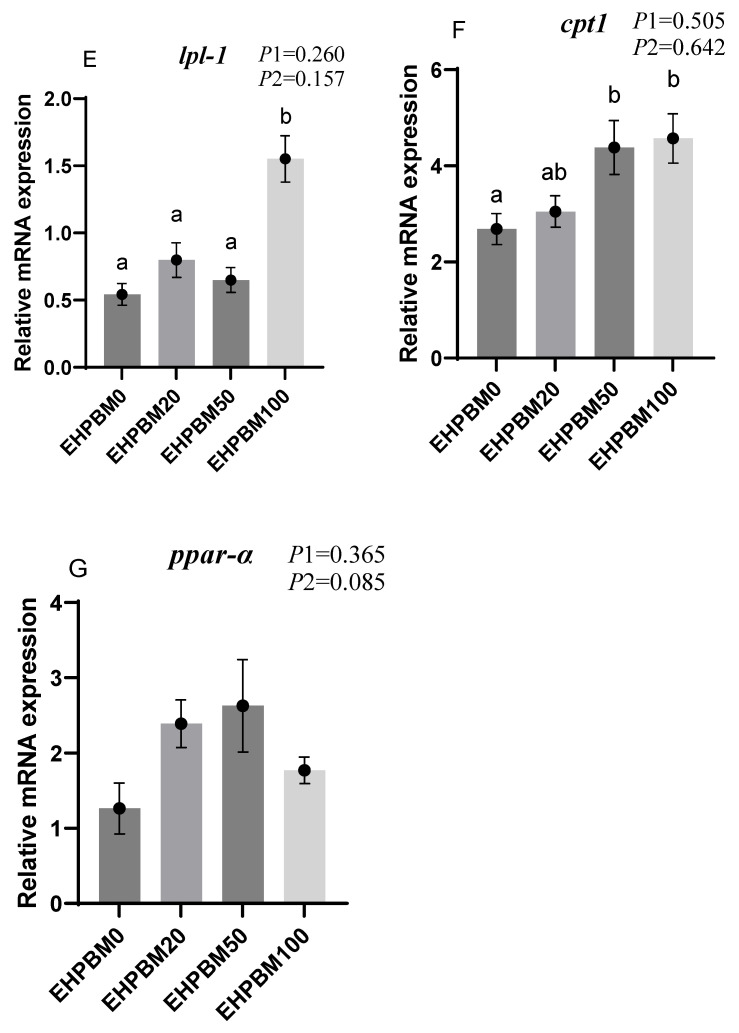
Expression of genes related to hepatic lipid metabolism. Note: Statistical significance between groups is indicated by differing superscripts. (**A**) *fabp1*; (**B**) *acc*; (**C**) *ppar-γ*; (**D**) *elovl2-l*; (**E**) *lpl-1*; (**F**) *cpt1*; (**G**) *ppar-α*. *p*1: the average of the *p*-values from the Shapiro–Wilk tests for each group; *p* > 0.05 indicates that the data follow a normal distribution. *p*2: *p*-value of the Levene’s test; *p* > 0.05 indicates that the data meet the assumption of homogeneity of variances.

Figure 4 presents the expression profiles of lipid metabolism-related genes under EHPBM substitution regimes. In the EHPBM100 group, significant upregulation was observed in *fabp1*, *lpl-1*, and *cpt1* expression compared with the EHPBM0 group (*p* < 0.05). Additionally, *cpt1* exhibited marked elevation even in the EHPBM 50 group compared with the EHPBM0 group (*p* < 0.05). Notably, EHPBM supplementation demonstrated no statistically significant correlations with the expression levels of *acc*, *ppar-γ*, *elovl2*, or *ppar-α* compared to controls (*p* > 0.05).

### 3.8. Histopathology of the Liver

**Figure 5:**
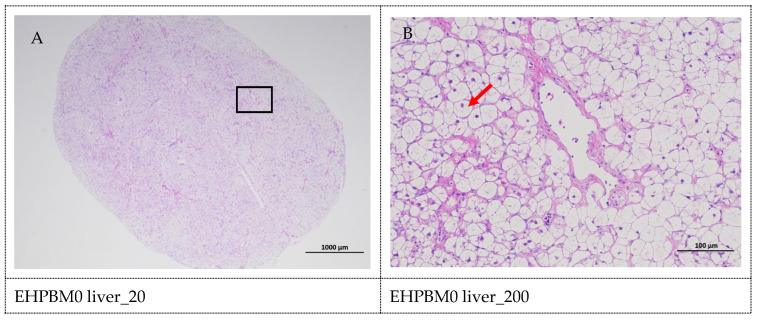

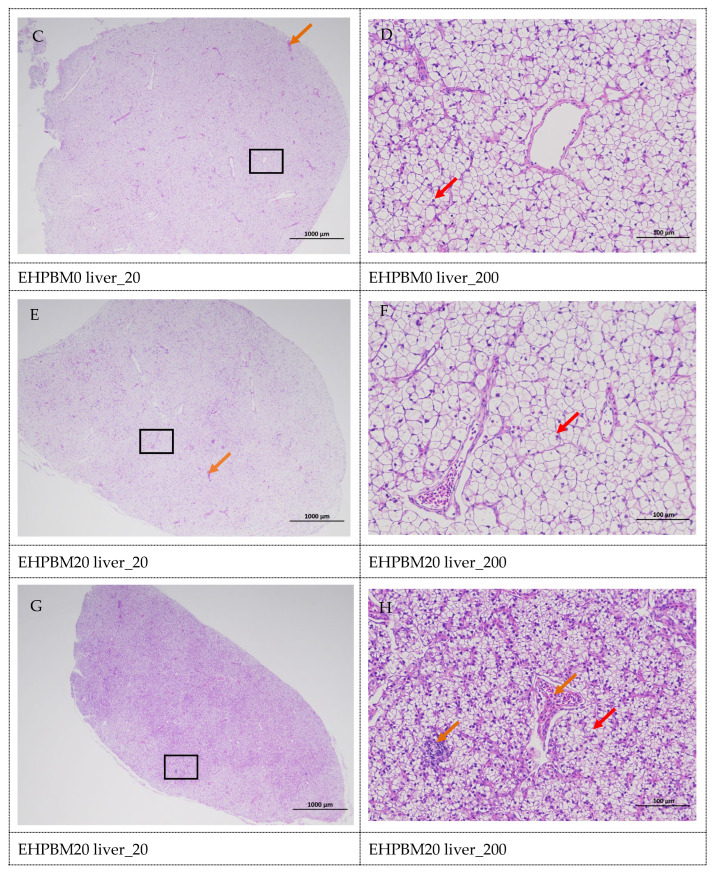

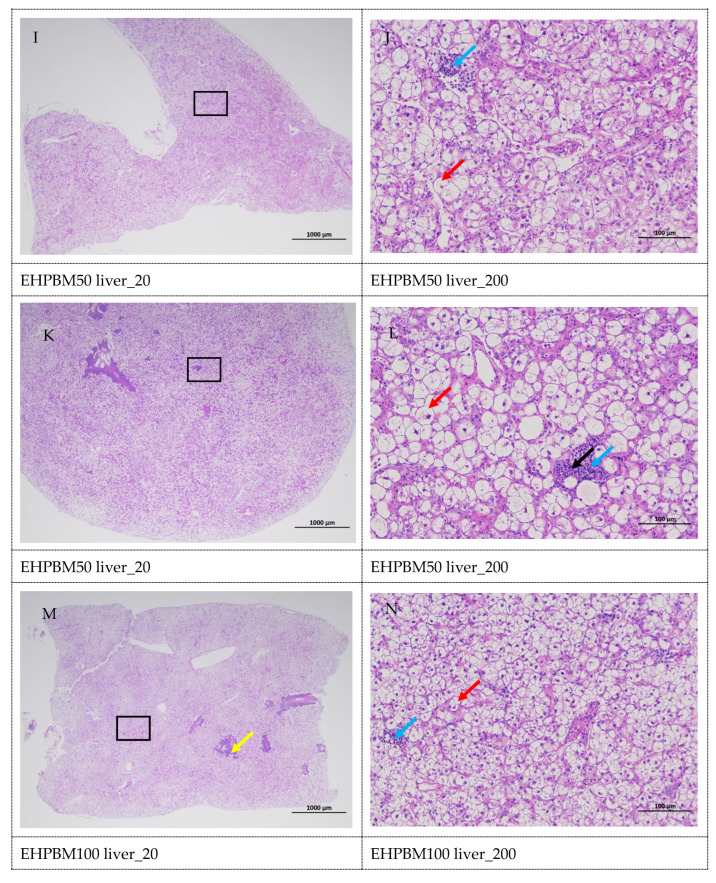

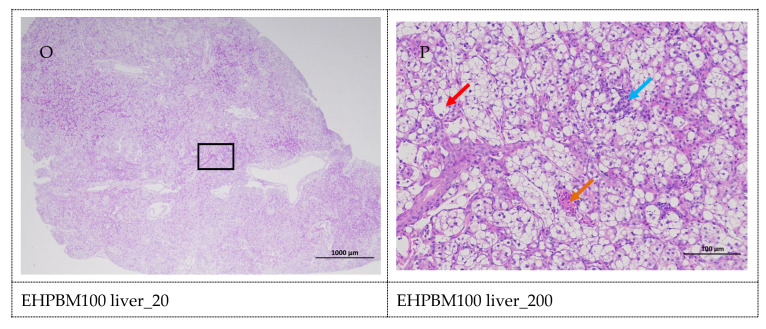
Liver pathology slide. Note: The black frame indicates the magnified area in the right image, the yellow arrow represents the presence of tubular acini, the red arrow represents cytoplasmic vacuolization of cells, the orange arrows indicate venous congestion, and the blue arrow represents focal inflammatory cell infiltration.

Figure 5 displays hepatic histopathological sections under different magnifications. The area enclosed by the black box on the left corresponds to the magnified region shown on the right. As shown, histopathological analysis of largemouth bass liver sections across four substitution levels demonstrated cytoplasmic vacuolization in hepatocytes from the EHPBM0 and EHPBM20 groups. In contrast, focal inflammatory infiltrates were observed at higher substitution groups (EHPBM50 and EHPBM100), with no evidence of advanced pathological lesions (e.g., necrosis, fibrosis) detected in any treatment group.

### 3.9. Intestinal Barrier and Transport Genes

**Figure 6:**
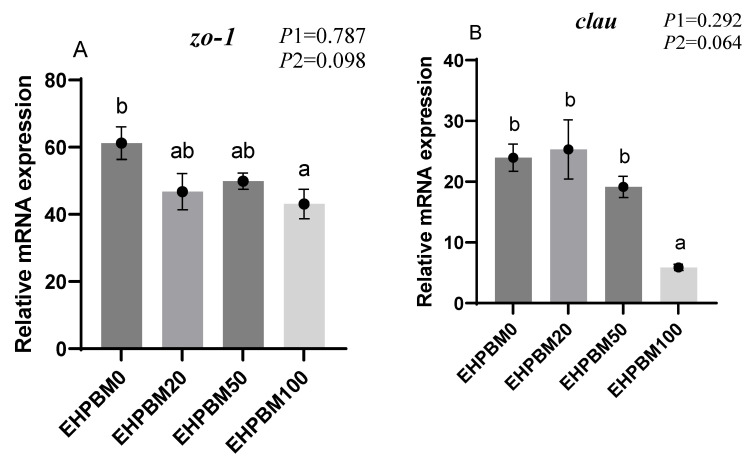

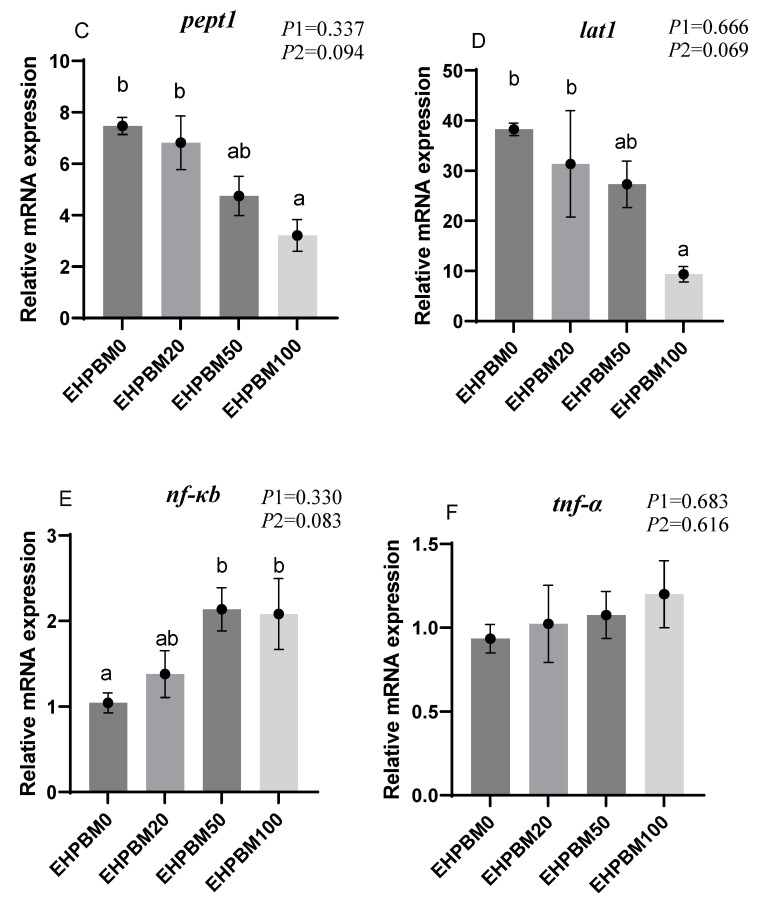
Expression of genes related to the intestinal barrier and transport. Note: Statistical significance between groups is indicated by differing superscripts. (**A**) zo-1; (**B**) clau; (**C**) pept1; (**D**) lat1; (**E**) nf-κb; (**F**) tnf-α. *p*1: the average of the *p*-values from the Shapiro–Wilk tests for each group; *p* > 0.05 indicates that the data follow a normal distribution. *p*2: *p*-value of the Levene’s test; *p* > 0.05 indicates that the data meet the assumption of homogeneity of variances.

Figure 6 illustrates the expression profiles of intestinal barrier and transport genes under varying EHPBM substitution levels. EHPBM100 group induced significant downregulation of *zo-1* and *clau* expression compared with the EHPBM0 group (*p* < 0.05). Concurrently, marked suppression was observed in *pept1* and *lat1* transcript levels compared with the EHPBM0 group (*p* < 0.05). A significantly increased expression of *nf-κb* can be observed in group EHPBM50 and EHPBM100 replacement levels in Figure 5E compared with the EHPBM0 group (*p* < 0.05). In contrast, EHPBM20 and EHPBM50 group substitution regimes exhibited no statistically significant effects on the expression of *zo-1*, *clau*, *pept1*, or *lat1* (*p* > 0.05).

## 4. Discussion

The findings of this study demonstrate that replacing 50% of fishmeal with EHPBM does not adversely affect the growth performance of largemouth bass. Previous studies have reported lower substitution thresholds for untreated meat and bone meal (MBM). For instance, MBM can replace 30% of fishmeal in cuneate drum (*Nibea miichthioides*) without compromising growth [48]; a 15% dietary protein substitution is feasible for Korean rockfish (*Sebastes schlegeli*) [49]; while olive flounder (*Paralichthys olivaceus*) tolerates up to 20% replacement without significant differences in condition factor (CF) and hepatosomatic index (HSI) [50]. However, higher substitution levels exhibit species-specific limitations: turbot (*Scophthalmus maximus* L.) shows reduced growth at 45% replacement [51], and juvenile giant trevally (*Caranx ignobilis*) experiences growth impairment at 25–50% substitution [52]. Notably, replacing 75–100% of fishmeal with a 1:1 MBM-protein concentrate mixture negatively affects the growth of climbing perch (*Anabas testudineus*) [53]. The enzymatic hydrolysis process likely enhances nutrient digestibility and absorption. Short peptides generated by enzymatic hydrolysis can be rapidly and efficiently absorbed by the intestine without requiring prior pancreatic digestion [54,55]. This may explain why a 50% replacement of fishmeal with untreated MBM significantly depressed growth performance in largemouth bass [22], whereas the EHPBM50 group in the present study maintained normal growth. However, when EHPBM replaced 100% of fishmeal in the diet, significant reductions in growth performance and increases in feed conversion ratio (FCR) were observed. Feed intake (FI) in the EHPBM100 group increased significantly. A similar response was observed in the GIFT strain of Nile tilapia (*Oreochromis niloticus*) when fishmeal was completely replaced [56], where both FI and feed conversion ratio (FCR) rose markedly, likely due to the low digestibility of the full substitution diet. Studies have shown that high ash content in feed negatively affects digestibility in largemouth bass [21] and rainbow trout (*Oncorhynchus mykiss*) [57]. In the present study, although the EHPBM100 diet had reduced total ash content, its lower digestible ash content [58] may have contributed to the impaired nutrient digestibility and absorption in this group. This provides a plausible explanation for the significant growth reduction and elevated FCR and FI observed in the EHPBM100 group.

Whole-body composition analysis revealed distinct species-specific responses to EHPBM substitution. While 50% replacement with enzymatically hydrolyzed MBM significantly reduced ash content in largemouth bass without affecting crude lipid or protein levels, contrasting patterns were observed in other species. Cuneate drum maintained stable body composition at 30% MBM substitution [48], Korean rockfish showed unaffected whole-body parameters at 15% replacement [49], and olive flounder exhibited comparable metrics with 10% MBM inclusion [50]. Ash content is closely linked to mineral composition [58]. The significant reduction in ash content at 50% and 100% EHPBM substitution levels suggests that unabsorbed minerals were likely excreted in feces, thereby decreasing whole-body ash retention. This aligns with previous findings that fishmeal replacement with animal protein hydrolysates significantly reduces ash content in largemouth bass [59]. At the 100% substitution level, whole-body crude lipid content decreased significantly while moisture content increased. This may result from the mobilization of endogenous lipid reserves for energy production [60], as fat mobilization promotes cellular hydration [61]. Similar patterns have been reported in Korean rockfish [49] and olive flounder [50].

The mechanistic target of rapamycin (mTOR) is a protein kinase that regulates cellular metabolism and growth [62], playing a key role in nutrient sensing [63]. Compared with the EHPBM0 group, the EHPBM50 group showed significantly upregulated expression of *mtor* and *rps6k*, whereas the EHPBM100 group exhibited significant upregulation of *4ebp1*. As a downstream effector of mTOR, RPS6K participates in regulating cell growth, proliferation, and metabolism [64]. These molecular results indicate that the EHPBM50 group maintained normal protein deposition and cell growth, whereas the EHPBM100 group showed impaired protein deposition. Studies have shown that protein synthesis is suppressed during the acute-phase response (APR) [65]. APR represents a universal physiological reaction to abnormal conditions such as tissue injury or infection [66], serving to re-establish homeostasis during inflammatory processes [67]. This response is typically induced by pro-inflammatory cytokines such as *tnf-α* [65]. The elevated expression of *tnf-α* in the EHPBM100 group may indicate the occurrence of APR. This possibility is further supported by the observed infiltration of inflammatory cells in liver pathological sections, suggesting the presence of mild hepatic inflammation in this group, which likely triggered the APR. The associated reduction in protein synthesis in the EHPBM100 group was evidenced by the dephosphorylation of 4EBP1. Furthermore, inflammatory conditions can adversely affect nutrient absorption and utilization. Additionally, the absence of certain bioactive compounds originally present in fishmeal in the EHPBM100 formulation may have further contributed to the reduced protein deposition and impaired growth performance.

*Fbp1* and *g6pase* encode key enzymes in the gluconeogenic pathway of animals [68]. In the EHPBM50 group, the expression of both genes was significantly down-regulated, indicating that EHPBM inclusion is associated with suppressed gluconeogenesis in largemouth bass. Glycolysis represents the primary catabolic pathway for glucose degradation in organisms [68]. The significant upregulation of the *pk* gene at the 50% EHPBM substitution level suggests enhanced glycolytic activity. This aligns with previous findings that MBM substitution reduces glucose anabolism in turbot [51]. Meanwhile, EHPBM supplementation did not significantly affect serum glucose (GLU) levels, indicating that EHPBM does not disrupt blood glucose homeostasis in largemouth bass.

When the replacement level of EHPBM for fishmeal exceeded 50%, the expression of *cpt1* and *lpl* was significantly up-regulated. This suggests that EHPBM may enhance lipid catabolism by promoting bile secretion. Studies have shown that dietary inclusion of rendered animal byproducts stimulates the production of bile, pancreatic enzymes, and other digestive secretions in fish [69]. Bile plays a crucial role in lipid digestion [70] and facilitates the elimination of cholesterol and toxic metabolites [71]. The 50% and 100% fishmeal replacement levels likely stimulated bile secretion in largemouth bass, thereby promoting lipid decomposition. This provides a plausible mechanism for the elevated *cpt1* expression observed in the EHPBM50 and EHPBM100 groups. Separate research in Florida pompano (*Trachinotus carolinus*) demonstrated that feeding poultry by-product meal significantly reduced serum bile acid and cholesterol levels [72]. Since bile salts emulsify cholesterol and promote its hepatic excretion [73], this may further explain the decreases in serum total cholesterol (TC) and triglycerides (TG) observed in the present study.

The excessive production and accumulation of reactive oxygen species (ROS) can induce cellular damage [74] and are associated with various inflammatory processes [75]. In the present study, all EHPBM substitution groups showed significantly enhanced activities of superoxide dismutase (SOD) and catalase (CAT), indicating that replacing fishmeal with EHPBM improves the antioxidant capacity of largemouth bass. Glutathione (GSH) plays an essential role in the antioxidant defense system [76]. The results revealed that 100% EHPBM substitution significantly increased tissue GSH concentration, while higher substitution levels (50% and 100%) notably elevated glutathione peroxidase (GSH-Px) activity. These findings suggest that elevated EHPBM inclusion enhances, rather than compromises, the stress resistance of largemouth bass. As malondialdehyde (MDA) serves as a direct marker of lipid peroxidation, its reduction typically reflects enhanced enzymatic antioxidant activity [77]. Significantly decreased MDA content was observed across all EHPBM substitution groups, collectively indicating improved antioxidant capacity in largemouth bass fed EHPBM-based diets. This enhancement may be attributed to antioxidant peptides released during enzymatic hydrolysis, which are known to exhibit free radical-scavenging activities [78]. Histopathological examination of liver sections confirmed that the improved antioxidant capacity was not accompanied by hepatocellular necrosis or fibrosis. This observation was further supported by the significantly reduced serum levels of alanine aminotransferase (ALT) and aspartate aminotransferase (AST) in the EHPBM50 and EHPBM100 groups. Although ALT and AST are established indicators of liver injury [79], they are also involved in metabolic processes [42,80]. The absence of hepatic lesions in EHPBM-fed fish suggests that the reduced ALT and AST levels may reflect modulated metabolic activity rather than liver damage. This interpretation is consistent with a previous study in juvenile giant trevally, where 50% fishmeal replacement with MBM similarly reduced serum ALT and AST levels [52].

Small peptides produced by enzymatic proteolysis exhibit enhanced nutritional value and superior gastrointestinal absorption efficiency [81]. As the primary site for bioactive peptide uptake [82], intestinal epithelial cells absorb these digestive products through specific transporter proteins [54]. Therefore, maintaining intestinal health is essential for optimal nutrient assimilation. Tight junction proteins function as selective physical barriers, providing a crucial defense against antigen and pathogen invasion [83]. Compromise of this barrier integrity plays a vital role in the development and progression of intestinal inflammation [84]. In the present study, 100% replacement of fishmeal with EHPBM significantly downregulated the gene expression of *zo-1* and *claudin*, indicating impaired intestinal barrier function. This suggests that high substitution levels may induce intestinal inflammation in largemouth bass. Meanwhile, the significantly elevated expression of *nf-κb* in both the EHPBM50 and EHPBM100 groups suggests the potential presence of a mild inflammatory response. This upregulation may represent a defensive mechanism in largemouth bass under high substitution conditions. A correlation between the di-/tri-peptide transporter *pept1* and intestinal inflammation has been demonstrated in murine models [85]. Thus, the inflammatory state in the EHPBM100 group may explain the observed downregulation of *pept1* and *lat1* gene expression. In contrast, a study on juvenile turbot (*Scophthalmus maximus* L.) reported that fishmeal replacement with MBM did not lead to reduced *pept1* expression [86], a discrepancy that may be attributed to interspecific differences or variations in physiological status.

## 5. Conclusions

In summary, enzymatically hydrolyzed pork bone meal (EHPBM) can effectively replace up to 50% of fishmeal in largemouth bass feed without negatively affecting growth performance. EHPBM supplementation significantly enhanced the systemic antioxidant capacity and did not induce adverse effects on liver health. However, excessive substitution levels may impair intestinal barrier function. These findings provide a scientific basis for the practical application of EHPBM in feed formulations and feeding strategies for largemouth bass.

## Figures and Tables

**Table 1:** Ingredient and nutrient composition of the experimental feed (% air dry basis).

Ingredients (%)	EHPBM0	EHPBM20	EHPBM50	EHPBM100
Fish meal ^a^	30.00	24.00	15.00	0.00
EHPBM ^b^	0.00	7.30	18.40	36.70
Soy concentrated protein ^a^	15.60	15.60	15.60	15.60
Wheat gluten	5.00	5.00	5.00	5.00
Swine blood meal	5.00	5.00	5.00	5.00
Chicken meal	12.00	12.00	12.00	12.00
Fish oil	4.10	4.20	4.50	4.90
Soybean oil	3.00	3.00	3.00	3.00
Wheat meal ^a^	5.50	5.50	5.50	5.50
Tapioca starch meal	6.00	6.00	6.00	6.00
Microcrystalline cellulose	4.70	4.40	2.90	0.00
Zeolite	3.90	2.60	1.30	0.00
Calcium dihydrogen phosphate	2.50	2.50	2.50	2.50
Mineral and Vitamin premix ^c^	2.00	2.00	2.00	2.00
Choline chloride	0.50	0.50	0.50	0.50
Vitamin C	0.10	0.10	0.10	0.10
Mold inhibitor	0.05	0.05	0.05	0.05
Antioxidant	0.05	0.05	0.05	0.05
L-Lysine ^d^	0.00	0.13	0.34	0.68
DL-Methionine ^d^	0.00	0.09	0.22	0.44
Analyzed composition				
Crude protein (%)	47.56	47.84	47.92	48.08
Crude lipid (%)	11.11	11.14	11.25	11.74
Ash (%)	14.41	12.90	11.91	10.02
Energy (KJ/g)	17.95	17.82	17.62	17.43

^a^ Fish meal, supplied by Wuxi Tongwei Feedstuffs Co., Ltd., Wuxi, China; wheat flour, supplied by Wuxi Tongwei Feedstuffs Co., Ltd., Wuxi, China; soy concentrated protein, supplied by Wuxi Tongwei Feedstuffs Co., Ltd., Wuxi, China; ^b^ EHPBM, supplied by JIANGSU MASUN BIO-TECH., LTD (Wuxi, China); ^c^ Mineral and Vitamin premix, supplied by HANOVE Animal Health Products (Wuxi, China). ^d^ DL-Methionine and L-lysine supplied by Feeer Co., Ltd. (Shanghai, China).

**Table 2:** Ingredient and nutrient composition of the fish meal and EHPBM (% air dry basis).

Ingredients (%)	Fish Meal	EHPBM
Crude protein	65.60	53.00
Crude lipid	9.50	4.00
Methionine	1.85	0.34
Lysine	5.05	2.35
Threonine	2.74	1.56

**Table 3:** Primer sequences.

Genes ^a^	(5′-3′) Forward Sequence	(5′-3′) Reverse Sequence	GenBank
*gapdh*	ACTGTCACTCCTCCATCTT	CACGGTTGCTGTATCCAA	AZA04761.1
Hepatic protein metabolism			
*mtor*	TTTGGAACCAAACCCCGTCA	ATCAGCTCACGGCAGTATCG	XM_038723321.1
*rps6k*	TCCAGAGACTCGTGACACCT	AGCTTGGCATACTCTGAGGC	XM_038713349.1
*4ebp1*	CCAGGATCATCTATGACGAAAG	TGCAGCGATATTGTTGTTGTTC	XM_038703879.1
*igf-1*	CCTCTGCCTGTGTATAATCA	TGTCCGTCTTAGCCATCT	[45]
Hepatic glucose metabolism			
*glut2*	GTGTTTGCTGTGCTGCTCCT	GCTCCGTATCGTCTTTGGG	[46]
*g6pase*	ACACAGCAGCCCTGTTCTAC	CCGTTCACACAGTACGGGAT	XM_038735542.1
*fbp1*	GCGATTGGCGAATTTATC	ACTCTGTGACGGCGGGTT	[47]
*pepck*	GGCAAAACCTGGAAGCAAGG	ATAATGGCGTCGATGGGGAC	MT431525.1
*pk*	CACGCAACACTGGCATCATC	TCGAAGCTCTCACATGCCTC	MT431526.1
*pfkfb2*	GGTGGCACTGGAAGATGTCA	TGGTGGCATCAAAAACAGCG	XP_018556727.1
*g6pdh*	ATGTGTCGCAGGAATGAG	TGATGAAGAAGAGGTGTGAA	XP_010777945.
Hepatic lipid metabolism			
*fabp1*	CTGGAGACTATTACTGGAGAG	ACACAATGCCACCAAGAG	Cluster-21914.4188
*acc*	TTACATCGCAGCCAACAG	CTCTCCACCTTCCTCTACA	XP_022609673.1
*elovl2-l*	GGACACAACAATACAAGATGG	GAACAGGTAGCACAGCAAT	Cluster-21914.20999
*lpl-1*	CTCCGCAGCCTACACTAA	CCAGCAGATGAATCCTCTC	FJ436090.1
*cpt1*	TTACCGTATGGCTATGACTG	GGCTCCGATAACACCTCT	XP_027141042.1
*ppar-α*	AGGCTTCATCACCAGAGA	TCCGCAGCAGATAATAGTAG	MK614719.1
*ppar-γ*	GAGTTCTCAGTCAAGTTCAAC	AATGTAGCACCGTCTCCT	MK614721.1
Intestinal barrier and transport			
*zo-1*	ATCTCAGCAGGGATTCGACG	CTTTTGCGGTGGCGTTGG	XM_038701018.1
*clau*	CCAGGGAAGGGGAGCAATG	GCTCTTTGAACCAGTGCGAC	XM_038713307.1
*pept1*	CCTATTTGCCTCGCTTTTGGTTGC	CATTAACCTTCGCCGTGAATGGG	MZ773078.1
*lat1*	CGCTGCCGAACCCATTTTTG	TTGAGCGTGAGCGTCTTTGT	XM_038706332.1
*nf-κb*	AGAAGACGACTCGGGGATGA	GCTTCTGCAGGTTCTGGTCT	XM_038699793.1
*tnf-α*	CTTCGTCTACAGCCAGGCATCG	TTTGGCACACCGACCTCACC	XM_038710731.1

^a^ *gapdh*, glyceraldehyde-3-phosphate dehydrogenase; *mtor*, mechanistic target of rapamycin; *rps6k*, ribosomal protein s6 kinase; *4ebp1*, eukaryotic translation initiation factor 4e-binding protein 1; *igf-1*, insulin-like growth factor 1; *glut2*, glucose transporter type 2; *g6pase*, glucose-6-phosphatase; *fbp1*, fructose-1,6-bisphosphatase 1; *pepck*, phosphoenolpyruvate carboxykinase; *pk*, pyruvate kinase; *pfkfb2*, 6-phosphofructo-2-kinase; *g6pdh*,glucose-6-phosphate dehydrogenase; *fabp1*, fatty acid binding protein 1; *acc*, acetyl-coa carboxylase; *elovl2-l*, elongation of very long chain fatty acids protein 2-like; *lpl-1*, lysophospholipase 1; *cpt1*, carnitine palmitoyltransferase 1; *ppar-α*, peroxisome proliferator-activated receptor alpha; *ppar-γ*, peroxisome proliferator-activated receptor gamma; *zo-1*, zonula occludens-1; *clau*, claudin; *pept1*, peptide transporter 1; *lat1*, l-type amino acid transporter 1; *nf-κb*, nuclear factor kappa-light-chain-enhancer of activated b cells; *tnf-α*, tumor necrosis factor-alpha.

**Table 4:** Growth performance.

Indexes	EHPBM0	EHPBM20	EHPBM50	EHPBM100	*p*1	*p*2
IBW (g)	6.61 ± 0.03	6.60 ± 0.03	6.59 ± 0.03	6.62 ± 0.03	0.348	0.791
FBW (g)	40.40 ± 1.11 ^b^	37.35 ± 0.46 ^b^	37.13 ± 0.73 ^b^	29.66 ± 1.15 ^a^	0.488	0.369
FCR	0.81 ± 0.02 ^a^	0.83 ± 0.02 ^a^	0.85 ± 0.01 ^a^	1.01 ± 0.03 ^b^	0.489	0.117
WGR (%)	511.24 ± 17.35 ^b^	465.68 ± 5.30 ^b^	463.18 ± 9.46 ^b^	348.38 ± 19.28 ^a^	0.429	0.276
SGR (%/days)	3.35 ± 0.05 ^b^	3.21 ± 0.02 ^b^	3.20 ± 0.03 ^b^	2.77 ± 0.08 ^a^	0.443	0.175
SR (%)	92.50 ± 2.50	98.75 ± 1.25	91.25 ± 4.27	93.75 ± 3.15	0.315	0.345
FI	2.15 ± 0.03 ^a^	2.17 ± 0.02 ^a^	2.20 ± 0.01 ^a^	2.36 ± 0.02 ^b^	0.505	0.487

Note: FCR = Weight of feed consumed/weight gain of fish(g); IBW(g) = sum of initial body weight/number of each cage; WGR(%) = 100 × (final body weight (g) − initial body weight(g))/initial body weight(g); FBW(g) = sum of final body weight/number in the end; SGR(%/d) = 100 × (Ln fish final weight(g)—Ln fish initial weight(g))/(days). ^ab^ Different groups with significant differences are represented by different letters, different groups without significant differences are represented by the same later, and no letter means that there is no significant difference between all groups. *p*1: the average of the *p*-values from the Shapiro–Wilk tests for each group; *p* > 0.05 indicates that the data follow a normal distribution. *p*2: *p*-value of the Levene’s test; *p* > 0.05 indicates that the data meet the assumption of homogeneity of variances.

**Table 5:** Whole body composition.

Indices	EHPBM0	EHPBM20	EHPBM50	EHPBM100	*p*1	*p*2
Moisture (%)	71.88 ± 0.18 ^a^	71.96 ± 0.04 ^a^	72.80 ± 0.34 ^ab^	73.46 ± 0.27 ^b^	0.653	0.248
Lipid (%)	7.37 ± 0.06 ^b^	7.38 ± 0.07 ^b^	6.57 ± 0.35 ^ab^	5.89 ± 0.31 ^a^	0.592	0.090
Protein (%)	17.07 ± 0.07	16.82 ± 0.09	16.91 ± 0.20	17.09 ± 0.24	0.510	0.150
Ash (%)	2.85 ± 0.06 ^b^	2.78 ± 0.20 ^ab^	2.23 ± 0.15 ^a^	2.24 ± 0.08 ^a^	0.587	0.142

Note: Values in the same column with different superscripts are significantly different (*p* < 0.05). *p*1: the average of the *p*-values from the Shapiro–Wilk tests for each group; *p* > 0.05 indicates that the data follow a normal distribution. *p*2: *p*-value of the Levene’s test; *p* > 0.05 indicates that the data meet the assumption of homogeneity of variances.

**Table 6:** Hepatic antioxidant indices.

Indexes	EHPBM0	EHPBM20	EHPBM50	EHPBM100	*p*1	*p*2
SOD (U/mgprot)	20.55±0.98 ^a^	80.63±2.11 ^b^	74.40±2.40 ^b^	89.11±1.62 ^c^	0.588	0.311
CAT (U/mgprot)	322.04±6.11 ^a^	425.35±15.57 ^b^	464.71±14.09 ^b^	1622.60±11.68 ^c^	0.502	0.544
MDA (nmol/mgprot)	1.48±0.08 ^b^	0.94±0.06 ^a^	1.11±0.06 ^a^	1.07±0.12 ^a^	0.565	0.083
GSH (μmol/gprot)	117.31±4.53 ^a^	129.58±7.76 ^a^	121.23±3.17 ^a^	173.65±8.71 ^b^	0.670	0.404
GSH-Px (U/mgprot)	26.70±1.80 ^a^	39.25±1.54 ^a^	129.11±3.58 ^b^	133.98±6.60 ^b^	0.178	0.099

Note: Values in the same column with different superscripts are significantly different (*p* < 0.05). *p*1: the average of the *p*-values from the Shapiro–Wilk tests for each group; *p* > 0.05 indicates that the data follow a normal distribution. *p*2: *p*-value of the Levene’s test; *p* > 0.05 indicates that the data meet the assumption of homogeneity of variances.

## Data Availability

Data are contained within the article.
